# Transgenic zebrafish as a model for investigating diabetic peripheral neuropathy: investigation of the role of insulin signaling

**DOI:** 10.3389/fncel.2024.1441827

**Published:** 2024-09-24

**Authors:** Dong-Won Lee, Hae-Chul Park, Dong Hwee Kim

**Affiliations:** ^1^Core Research and Development Center, Korea University Ansan Hospital, Ansan, Republic of Korea; ^2^Zebrafish Translational Medical Research Center, College of Medicine, Korea University, Ansan, Republic of Korea; ^3^Department of Biomedical Sciences, College of Medicine, Korea University, Ansan, Republic of Korea; ^4^Department of Physical Medicine and Rehabilitation, College of Medicine, Korea University, Ansan, Republic of Korea

**Keywords:** diabetic peripheral neuropathy, chemogenetic ablation, zebrafish disease model, insulin signaling, synapse formation

## Abstract

Diabetic peripheral neuropathy (DPN), a complication of diabetes mellitus (DM), is a neurodegenerative disorder that results from hyperglycemic damage and deficient insulin receptor (IR) signaling in peripheral nerves, triggered by failure of insulin production and insulin resistance. IR signaling plays an important role in nutrient metabolism and synaptic formation and maintenance in peripheral neurons. Although several animal models of DPN have been developed to identify new drug candidates using cytotoxic reagents, nutrient-rich diets, and genetic manipulations, a model showing beneficial effects remains to be established. In this study, we aimed to develop a DPN animal model using zebrafish to validate the effects of drug candidates on sensory neuropathy through in vivo imaging during the early larval stage. To achieve this, we generated *Tg (ins:gal4p16);Tg (5uas:epNTR-p2a-mcherry)* zebrafish using an enhanced potency nitroreductase (epNTR)-mediated chemogenetic ablation system, which showed highly efficient ablation of pancreatic β-cells following treatment with low-dose metronidazole (MTZ). Using in vivo live imaging, we observed that sensory nerve endings and postsynaptic formation in the peripheral lateral line (PLL) were defective, followed by a disturbance in rheotaxis behavior without any locomotory behavioral changes. Despite defects in sensory nerves and elevated glucose levels, both reactive oxygen species (ROS) levels, a primary cause of DPN, and the number of ganglion cells, remained normal. Furthermore, we found that the activity of mTOR, a downstream target of IR signaling, was decreased in the PLL ganglion cells of the transgenic zebrafish. Our data indicates that peripheral neuropathy results from the loss of IR signaling due to insulin deficiency rather than hyperglycemia alone.

## 1 Introduction

Diabetic mellitus (DM) is a metabolic disorder characterized by the inadequate production of insulin hormone by pancreatic β-cells or the inability of insulin-accepting cells to respond effectively. This results in an increase in the blood sugar levels, which leads to cellular damage. Hyperglycemia results in several complications, such as peripheral neuropathy, cardiomyopathy, and nephropathy. Diabetic peripheral neuropathy (DPN), a neurodegenerative disorder characterized by the presence of hyperglycemic damage in the peripheral nerves owing to deficient insulin receptor (IR) signaling, is a common complication of DM (Kim and Feldman, 2012). Several animal models of DPN, such as streptozotocin, high-fat diet, and genetic manipulation, have been created to identify new drug candidates (Leiter, 2005; Obrosova et al., 2007; Vincent et al., 2009; Alpers and Hudkins, 2011); however, drugs showing beneficial effects on DPN remain to be established. Thus, an improved DPN disease model that facilitates further verification of drug efficacy and its functional role is required.

IR signaling is an intracellular signaling pathway that regulates glucose metabolism in the peripheral tissues, such as the liver, muscle, and adipose tissue. It plays a crucial role in maintaining neuronal health and synapse formation in the nervous system (Wan et al., 1997; Lee et al., 2005; Chiu et al., 2008). IRs are predominantly expressed in small nociceptive dorsal root ganglion (DRG) neurons in the peripheral nervous system (PNS) (Sugimoto et al., 2000; Sugimoto et al., 2002; Shettar and Muttagi, 2012), and their stimulation augments neuritogenesis of chick sympathetic and DRG sensory neurons via the activation of the PI3-K/AKT pathway (Huang et al., 2005). Thus, insulin and its signaling pathway function as neurotrophic supporters of PNS. Loss of IR signaling due to insulinopenia (type 1) without hyperglycemia has been strongly implicated in different phenotypes of DPN, such as hyperalgesia, reduced nerve conduction velocity, and unmyelinated axonal loss in animal models (Murakawa et al., 2002; Romanovsky et al., 2006; Romanovsky et al., 2010). These findings indicate that IR signaling can cause signs of DPN.

The transparency of the live embryo of Zebrafish enables the visualization of real-time changes in the peripheral nerves in the disease state. Furthermore, its rapid development and permeability facilitate the assessment of the efficacy of pharmacological candidates for treating peripheral neuropathy during the early developmental period (Taylor et al., 2010). Thus, Zebrafish is a promising vertebrate model for evaluating the pathological mechanisms of peripheral neuropathy and drug testing. The peripheral lateral line nerve (PLLn), which results in rheotaxis behavior underneath the skin, enables the perception of water flow. Thus, PLLn can be used to analyze nerve injuries in vivo (Olszewski et al., 2012). Several types of models using paclitaxel and oxaliplatin have been developed to reveal the pathomechanism of peripheral neuropathy and validate the preventive effects of pharmaceutical drugs (Lisse et al., 2016; Cirrincione et al., 2020; Lee et al., 2024). Among them, zebrafish is a valuable biomedical model for evaluating peripheral neuropathy.

This study aimed to develop a DPN zebrafish model for the assessment of the effects of drugs on sensory neuropathy during the early larval stage. *Tg (ins:gal4p16);Tg (5uas:epNTR-p2a-mcherry)* transgenic zebrafish were generated to demonstrate the damage to the nerve endings in PLLn owing to deficiency of pancreatic β-cell. Synapse formation of the sensory nerve, insulin-related signaling pathway, and behavioral changes were evaluated subsequently.

## 2 Materials and methods

### 2.1 Zebrafish animal husbandry

*Tg (ins:gal4vp16);Tg (5uas:epNTR-p2a-mcherry)* (#FRZCC 1053) lines were established, and *Tg (tubb2b:dsred)* (#FRZCC 1064) transgenic lines of either sex were used in this study (Peri and Nusslein-Volhard, 2008). All adult and larval zebrafish were maintained in E3 embryo medium (EM) at 28.5°C (14 h light/ 10 h dark cycle). The developmental stages were determined based on the number of days post-fertilization (dpf) according to the morphological development criteria.

### 2.2 Generation of the *Tg (ins:gal4vp16); Tg (5uas:epNTR-p2a-mcherry)* line

The transgenic line was generated as follows. The transgene construct pTol2_ins:gal4vp16 was generated by inducing the LR reaction of gateway cloning using LR clonase (Invitrogen), p5E:ins [24], pME:gal4vp16, p3E:pA, and the pDestTol2CG2 plasmid [25]. The middle entry clone pME:epNTR was generated by inducing the BP reaction of gateway cloning with BP clonase (Invitrogen). The epNTR PCR product was amplified using attB1F_epNTR_F, attB2R_epNTR_R primers, and pDONR221 to generate the transgene pTol2_5uas:epNTR-p2a-mcherry construct. The pTol2_5uas:epNTR-p2a-mcherry construct was generated by inducing the LR reaction of Gateway cloning with LR clonase (Invitrogen), p5E:5uas, pME:epNTR, p3E:p2a-mcherry, and the pDestTol2CG2 plasmid. The following primers were used for Gateway cloning: attB1F_epNTR_F (5′-GGGGACAAGTTTGTACAAAAAAGCAGGCTCCATGGATATT ATTAGTGTGGCCCTGAAGAG-3′) and attB2R_epNTR_R (5′-GGGGACCACTTTGTACAAGAAAGCTGGGTCCGCCACCTC TGTCAGTGTGATGTTCTGA-3′).

The pTol2_ins:gal4vp16 and pTol2_5uas:epNTR-p2a-mcherry constructs were injected into one-cell fertilized embryo with transposase mRNA to generate *Tg (ins:gal4vp16)* and *Tg (5uas:epNTR-p2a-mcherry)* lines, respectively.

### 2.3 Drug preparation and administration

MTZ powder (0.043 g; Sigma, Cat no. #M1547, USA) was dissolved in 50 mL of EM and 100 μl of dimethyl sulfoxide (DMSO) solvent to obtain an MTZ solution of 5 mM concentration. The MTZ solution was administered to transgenic larvae without changing the solution daily from 3 to 9 dpf. BMS-754807 (10 mM; Selleck, Cat no. S1124, USA) was diluted in EM to 10 uM and administered to the transgenic larvae from 3 to 9 dpf. For all drug treatment experiments, 0.1% DMSO was used as control.

### 2.4 Quantitative RT-PCR (qRT-PCR) analysis

The insulin mRNA expression levels were analyzed via qRT-PCR using a LightCycler96 device (Roche Diagnostics) and FastStart SYBR Green Master (Roche Diagnostics). The following primer sequences were used to quantify the insulin mRNA levels: ins_qRT_F (5′-ATGGCAGTGTGGCTTCAGGC-3′) and ins_qRT_R (5′- CAGCCACCTCAGTTTCCTGG-3′).

### 2.5 Whole-mount immunohistochemistry

A modified version of whole-mount immunohistochemistry (WM-IHC) described in a previous study was performed [26]. The primary and secondary antibodies used in this study were as follows: mouse anti-MAGUK to mark the postysnapse (1:200; Neuromab, 75-029), rabbit anti-pS6 for mTOR activity (1:100; catalog, Cell Signaling Technology, #2215S), mouse anti-Dsred for mcherry labeling (1:200; TaKaRa, 632492), rabbit anti-Alexa Fluor 488, mouse anti-Alexa Fluor 568, and mouse anti-Alexa Fluor 647 (1:1,000, Molecular Probes).

### 2.6 Blood glucose measurement

Zebrafish larvae (*n* = 30) were rinsed thrice with EM, homogenized using a mortar and pestle, and centrifuged at 12,000 rpm for 1 min. The resulting supernatant was loaded onto a blood glucose meter (On Call Extra, ACON Laboratories Inc., CA, USA).

### 2.7 Staining and measurement of reactive oxygen species

CellROX (10 μM; Thermo, C10444) that bound ROS for oxidation was administered to the zebrafish larvae for 1 h and washed thrice with EM for 10 min. The fluorescence intensity of CellROX, used to measure ROS levels in PLLg, was determined using the NIS-Elements software program (Nikon).

### 2.8 In-vivo imaging

The zebrafish larvae were anesthetized by administering 0.17 mg/mL tricaine (Sigma, Cat No. #A5040) in EM solution and were embedded on their lateral side in 1.5% low-melt agarose containing tricaine. Fluorescence z-stack images of the embedded zebrafish larvae were acquired using an A1Si confocal microscope (Nikon).

### 2.9 Behavior analysis

The locomotion behavior test was performed using the EthoVision XT 12 system (Noldus, Wageningen, Netherlands). The zebrafish larvae were individually placed into 48-well plates containing 800 μL of EM, acclimated for 30 min, and recorded for 5 min under light conditions. The distance moved (mm) and the velocity (mm/s) were determined for the analysis. The rheotaxis behavior test was performed as described in a previous protocol (Olszewski et al., 2012). The zebrafish larvae were placed into an acrylic board-made observation chamber, acclimated for 30 min, and recorded using a video camera (Sony, Exmor RS) for 5 min, with water flowing at a constant velocity (84 mm/s). Images were manually captured every 30 s, from the video, and the angle of the larval body relative to the direction of water flow was measured using ImageJ (NIH).

Rheotaxis preference was analyzed based on the orientation of the larval body against constant water flow. Orientation of the larval body in any other position was defined as negative preference.

Locomotion and rheotaxis behavior were sequentially analyzed three independent times using a repeat-test.

### 2.10 Statistical analysis

Statistical analyses of the in vivo imaging and locomotion behavior data were performed using a two-tailed unpaired Student’s *t*-test and one-way analysis of variance (ANOVA), followed by Tukey’s multiple comparison test. All statistical analyses were performed using GraphPad Prism 9.0 (GraphPad Software, San Diego, CA, USA). Statistical data of the rheotaxis behavior test were analyzed using the chi-square test. Statistical significance was set at *p* < 0.05. Statistical significance was denoted as follows: non-significant (n.s.), *p < 0.05, **p < 0.01, ***p < 0.001, and ****p < 0.0001.

## 3 Results

### 3.1 MTZ induces ablation of pancreatic β-cells and inhibits insulin production in Ins:epNTR-mCherry zebrafish

**FIGURE 1:**
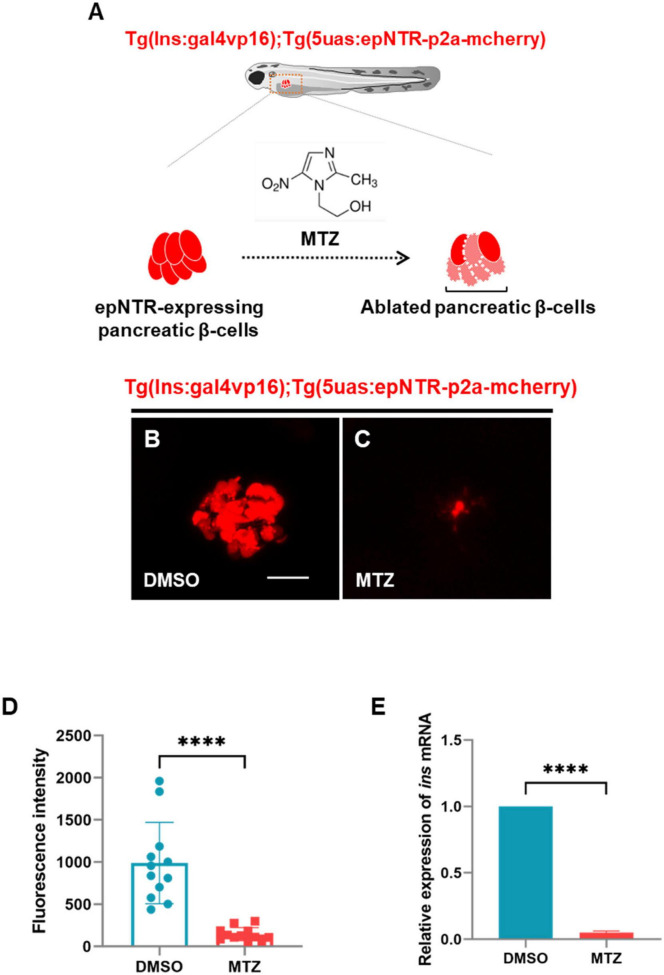
Ablation of pancreatic β-cells induced depletion of insulin production in ins:epNTR-mCherry zebrafish. **(A)** Schematics of the transgenic zebrafish showing the ablation of pancreatic β-cells using epNTR-mediated chemogenetic ablation system. **(B,C)** In-vivo fluorescence images showing pancreatic β-cells in Ins:epNTR-mCherry larvae treated with DMSO **(B)** and MTZ **(C)** at 9 dpf. **(D)** Quantification of fluorescence intensity in mcherry-expressing pancreatic β-cells from panel **(B)** and **(C)**, ****p < 0.0001, *t*-test (*n* = 12 larvae per group). **(E)** Quantification of expression of *ins* mRNA in transgenic larvae, ****p < 0.0001, *t*-test (*n* = 15 larvae per group). Scale bar, 20 μm in **(B)**.

To deplete insulin production to trigger damage to the nerve endings in the PLLn, we used an epNTR-mediated chemogenetic ablation system that can ablate specific targeted cell types via the conversion of MTZ to a cytotoxin by the epNTR enzyme to generate *Tg (ins:gal4p16);Tg (5uas:epNTR-p2a-mcherry)* zebrafish with selectively ablated pancreatic β-cells (Figure 1A; Tabor et al., 2014). Hereafter, we refer to this transgenic line as Ins:epNTR-mCherry. Through in-vivo imaging, we observed that the number of mcherry-expressing pancreatic β-cells in the MTZ-treated larvae were significantly lower than that in the DMSO-treated larvae (Figures 1B, C). Similarly, the intensity of mCherry fluorescence in the MTZ-treated larvae was significantly lower than that in the DMSO-treated larvae (Figure 1D). By using qRT-PCR, we further confirmed that the expression levels of insulin *(ins)* mRNA in the MTZ-treated larvae was significantly lower than that in the DMSO-treated larvae (Figure 1E), indicating that the pancreatic β-cells were ablated by MTZ in Ins:epNTR-mCherry larvae. These findings suggest that the depletion of pancreatic β-cells blocks insulin production in mTOR in the PLLg larvae.

### 3.2 Depletion of insulin production causes peripheral neuropathy in the PLLn in Ins:epNTR-mCherry zebrafish

**FIGURE 2:**
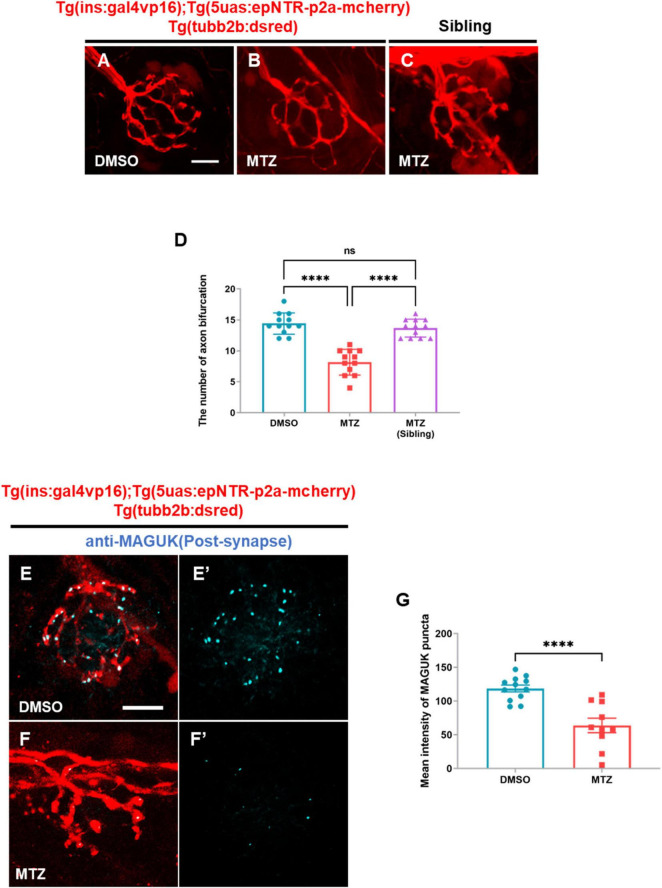
Depletion of insulin production caused peripheral neuropathy in the peripheral lateral line nerve of ins:epNTR-mCherry zebrafish. **(A–C)** *In-vivo* fluorescence images showing sensory nerve endings in Ins:epNTR-mCherry expressing *Tg (tubb2b:dsred)* larvae treated with DMSO **(A)** and MTZ **(B)** at 9 dpf. In-vivo fluorescence images showing sensory nerve endings in *Tg (tubb2b:dsred)* siblings treated with MTZ **(C)** at 9 dpf. **(D)** Quantification of the number of axon bifurcation from panel **(A–C)**, ****p < 0.0001, one-way ANOVA (*n* = 12 larvae per group). **(E–F’)** Fluorescence images showing sensory nerve endings with postsynaptic MAGUK^+^ puncta in Ins:epNTR-mCherry expressing *Tg (tubb2b:dsred)* larvae treated with DMSO **(E,E’)** and MTZ **(F,F’)** at 9 dpf. **(G)** Quantification of fluorescence intensity of MAGUK^+^ puncta in the transgenic larvae presented in panels **(E’)** and **(F’)**, ****p < 0.0001, *t*-test (*n* = 12 in DMSO group and *n* = 10 in MTZ group). Scale bar, 10 μm in **(A)** and **(E).**

To analyze peripheral neuropathy in the PLLn, we crossed *Tg (tubb2b:dsred)* expressing the dsred fluorescent protein in the PLLn with Ins:epNTR-mcherry and constructed a triple-transgenic line. Although expressing fluorescent proteins with similar emission wavelengths, this line did not express them in the same cell types, owing to their discrete regulation by the promoters in the transgenes (Supplementary Figure 1). Through in-vivo imaging, we observed that the number of axon bifurcations was markedly reduced in the MTZ-treated larvae compared with that in the DMSO-treated larvae (Figures 2A, B, D). The administration of MTZ to the larvae without epNTR expression had no effect on the number of sensory nerves (Figures 2C, D). This finding indicates that MTZ did not exert an off-target effect on the sensory properties of zebrafish. Using WM-IHC with an anti-postsynaptic MAGUK antibody that labeled postsynaptic MAGUK scaffold proteins in the postsynaptic terminals of the PLL nerve endings, we observed that the intensity of MAGUK^+^ synaptic puncta in PLL nerve endings in the MTZ-treated larvae was significantly lower than that in the DMSO-treated larvae, with the fluorescent signal remaining very weak in the PLL nerve endings (Figures 2E–G).

These data indicated that the depletion of pancreatic β-cells results in postsynaptic loss in Ins:epNTR-mCherry larvae, suggesting that the depletion of insulin production causes peripheral neuropathy in Ins:epNTR-mCherry larvae.

### 3.3 Depletion of insulin production results in defective rheotaxis behavior, but not locomotor behavior, in Ins:epNTR-mCherry zebrafish

**FIGURE 3:**
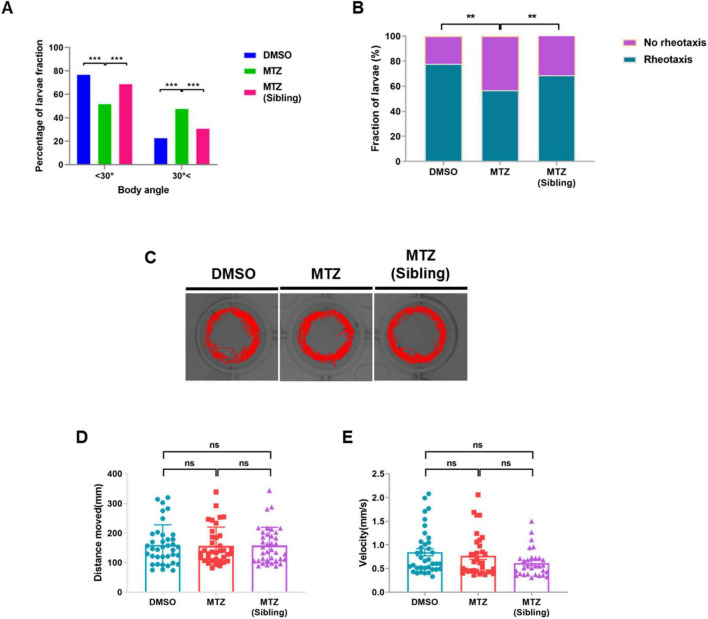
Depletion of insulin production results in defective rheotaxis behavior, but not locomotor behavior, in ins:epNTR-mCherry zebrafish. **(A)** Quantification of the distribution of the Ins:epNTR-mCherry larvae performing rheotactic angles following treatment with DMSO and MTZ, and siblings treated with MTZ at 9 dpf, ***p = 0.0004, Chi-squared test (*n* = 15 larvae per group). **(B)** Quantification of the percentage of the transgenic larvae showing rheotactic behavior following treatment with DMSO and MTZ, and siblings with MTZ at 9 dpf, **p = 0.0064, Chi-squared test (*n* = 15 larvae per group). **(C)** Tracking visualization of the transgenic larvae performing free locomotion in light state at 9 dpf. **(D,E)** Quantification of the distance moved (mm) **(D)** and velocity (mm/s) **(E)** from the transgenic larvae performing free locomotion under light conditions following treatment with DMSO and MTZ, and sibling with MTZ at 9 dpf (*n* = 32 larvae per group).

Previous reports have indicated that the PLL system is involved in transmitting sensory input from the PLLn endings to the central nervous system (CNS) for rheotaxis behavior in zebrafish (Olszewski et al., 2012). Zebrafish larvae showed a positive rheotactic response within 30° of body orientation for flow directions during streaming water flow, and no bias without water flow conditions (Suli et al., 2012). Therefore, we tested whether synaptic loss in PLLn endings was followed by rheotaxis behavioral changes. The rheotaxis behavior test revealed that the number of MTZ-treated larvae with a high rheotactic angle (> 30°) was higher than that of the DMSO-treated larvae and MTZ-treated siblings (Supplementary Movie 1 and Figure 3A). The fraction of larvae showing rheotactic behavior among the MTZ-treated larvae was lower relative to those among the DMSO-treated larvae and MTZ-treated siblings (Figure 3B). The locomotor behavior test revealed that the locomotor activities were similar in the MTZ-treated larvae, DMSO-treated larvae, and MTZ-treated siblings (Figures 3C–E). These findings indicate that the ablation of the pancreatic β-cells resulted in defective rheotaxis behavior in Ins:epNTR-mCherry larvae; however, the locomotor behavior remained unaffected, suggesting that the depletion of insulin production results in defective rheotaxis behavior in Ins:epNTR-mCherry larvae.

### 3.4 Depletion of insulin production results in elevated glucose levels, but not ROS, in Ins:epNTR-mCherry zebrafish

**FIGURE 4:**
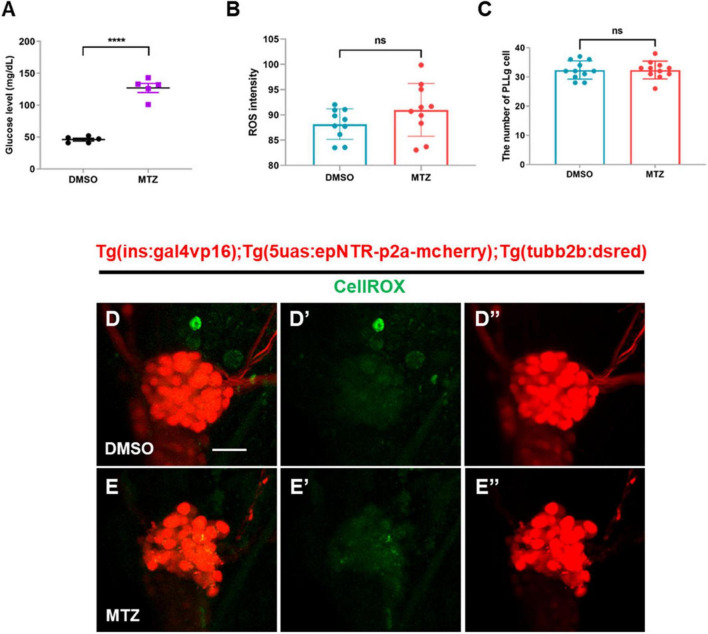
Depletion of insulin production elevates glucose levels, but not ROS, in ins:epNTR-mCherry zebrafish. **(A)** Quantification of the glucose levels (mg/dL) in Ins:epNTR-mCherry larvae treated with DMSO and MTZ at 9 dpf, ****p < 0.0001, *t*-test (*n* = 20 larvae per one data point, total *n* = 100 larvae per group). **(B)** Quantification of the ROS intensity in the PLLg cells of Ins:epNTR-mCherry expressing *Tg (tubb2b:dsred)* larvae presented in panels **(D’)** and **(E’)** (*n* = 10 larvae per group). **(C)** Quantification of the number of PLLg cells of the transgenic larvae presented in panels **(D”)** and **(E”)** (*n* = 11 larvae per group). **(D–E”)** *In-vivo* fluorescence images showing PLLg cell and CellROX ROS detector in the transgenic larvae treated with DMSO **(D–D”)** or MTZ **(E–E”)** at 9 dpf. Scale bar, 20 μm in **(D)**.

Previous studies have reported that low insulin production suppresses glucose uptake from blood vessels, subsequently resulting in hyperglycemia that could induce reactive oxygen species (ROS) production in peripheral neuron (Vincent et al., 2005). Therefore, we tested whether ablation of pancreatic β-cells induced hyperglycemia followed by ROS production in the PLL ganglions (PLLgs) in Ins:epNTR-mCherry larvae. The glucose levels in the MTZ-treated larvae were 2.5-fold higher than those in the DMSO-treated larvae (Figure 4A). However, the ROS intensity following CellROX staining observed in the MTZ-treated larvae did not differ from that observed in the DMSO-treated larvae, despite the increase in glucose levels in the absence of insulin production (Figures 4B, D’, E’). In addition, the number of PLLg in the MTZ-treated larvae did not differ from that in the DMSO-treated larvae (Figures 4C, D”, E”).

These findings indicate that the depletion of pancreatic β-cells elevates the glucose levels. However, this was not followed by the overproduction of ROS, indicating that hyperglycemia does not induce the overproduction of ROS in Ins:epNTR-mCherry larvae 9 dpf.

### 3.5 Inhibition of IR reduced the nerve endings of PLL in the zebrafish treated with BMS-754807

**FIGURE 5:**
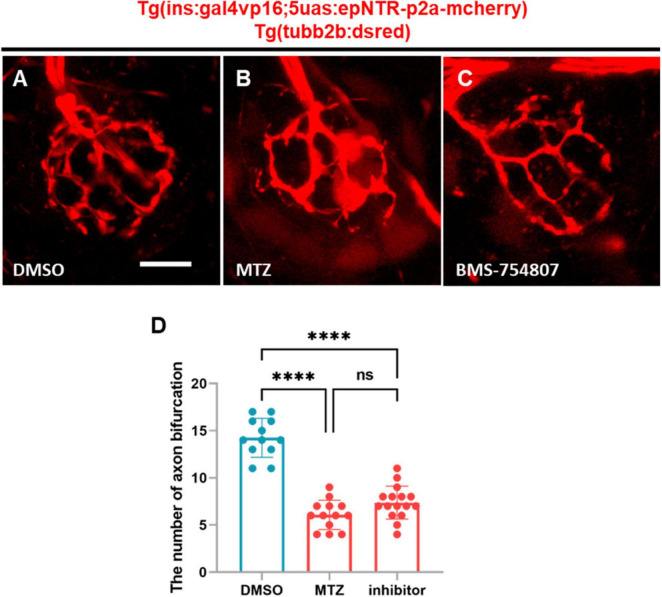
BMS-754807-induced IR inhibition reduces PLL nerve endings in ins:epNTR-mCherry zebrafish. **(A–C)**. In-vivo fluorescence images showing sensory nerve endings in Ins:epNTR-mCherry expressing *Tg (tubb2b:dsred)* larvae treated with DMSO **(A)**, MTZ, **(B)** and BMS-754807 **(C)** at 9 dpf. **(D)** Quantification of the number of axon bifurcation as depicted in panels **(A–C)**, ****p < 0.0001, one-way ANOVA (*n* = 12 larvae in the DMSO group, *n* = 13 larvae in the MTZ group, and *n* = 16 larvae in the inhibitor group). Scale bar, 10 μm in **(A)**.

It was previously reported that insulin hormone acted on both IR and IGF-1R to regulate diverse cellular processes, including nutrient metabolism and cell growth and differentiation, in peripheral tissues (Chang et al., 2004). Therefore, we treated *Tg (tubb2b:dsred)* zebrafish with BMS-754807, an inhibitor of both IR and IGF-1R, and subsequently analyzed the axon bifurcations in PLLn endings. We observed that the number of axonal bifurcations in the larvae treated with BMS-754807 was significantly lower than that in the DMSO-treated larvae (Figures 5A, B, D). The inhibition of IR did not result in any differences in the MTZ-treated larvae (Figures 5A, C, D), indicating that IR inhibition reduced the PLLn endings in zebrafish. These findings suggest that IR induces peripheral neuropathy in the PLL of Ins:epNTR-mCherry larvae.

### 3.6 Depletion of insulin production downregulates the insulin signaling pathway in PLLgs in Ins:epNTR-mCherry zebrafish

**FIGURE 6:**
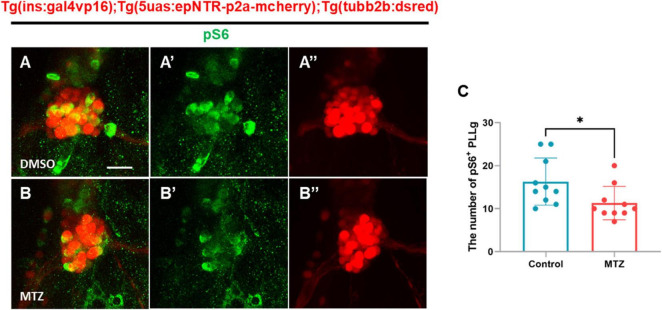
Depletion of insulin production downregulates the mTOR signaling pathway in the PLLg in ins:epNTR-mCherry zebrafish. **(A–B”)** Fluorescence images showing the PLLg and pS6^+^ cells in Ins:epNTR-mCherry expressing *Tg (tubb2b:dsred)* larvae treated with DMSO **(A–A”)** and MTZ **(B–B”)** at 9 dpf. **(C)** Quantification of the number of pS6^+^ PLLg cells from panels **(A)** and **(B)**, *p = 0.0305, *t*-test (*n* = 10 larvae per group). Scale bar, 20 μm in **(A)**.

Previous studies have demonstrated that mTOR signaling regulates dendritic branching and synapse formation in CNS neurons (Saltiel and Kahn, 2001). To investigate whether mTOR activity, a downstream target of IR signaling, was downregulated by the depletion of insulin production, we conducted immunohistochemical analysis using an anti-pS6 antibody, which marks ribosomal protein S6, which is phosphorylated and activated by mTOR complexes. Immunohistochemistry revealed that the number of pS6^+^ PLLg cells in the MTZ-treated larvae was lower than that in the DMSO-treated larvae (Figures 6A–C). This finding indicates that the depletion of insulin production decreases the activity of the insulin signaling pathway through mTOR in the PLLg in Ins:epNTR-mCherry larvae.

## 4 Discussion

The present study revealed that the depletion of insulin signaling in the established Ins:epNTR-mCherry zebrafish model resulted in peripheral neuropathy in the PLLn. This peripheral neuropathy was characterized by a loss of postsynaptic nerves and synapse and a reduction in mTOR signaling in the PLLg due to the depletion of insulin signaling. Thus, this model can be used to validate candidate drugs for the prevention of DPN.

Previous studies have reported deficient insulin production induced via the injection of chemicals such as STZ and alloxan and a genetic manipulation system in several types of zebrafish models. STZ and alloxan can effectively ablate pancreatic β-cells in adult zebrafish and its larvae, reduce the insulin expression levels, and elevate blood glucose levels. Although they present with stable hyperglycemic complications, such as retinopathy and nephropathy, they can also exhibit off-target mediated toxic effects (Watkins and Sherman, 1992; Diab et al., 2015; Radenkovic et al., 2016). A chemogenetic ablation system with NTR1.0/MTZ efficiently induces selective ablation of pancreatic β-cells, followed by the upregulation of blood glucose levels in zebrafish larvae (Rocker et al., 2019; Kim et al., 2021). However, this system requires the administration of a high dose of MTZ (10 mM), which exhibits near-toxic and potential off-target effects for sustained ablation (Cheng et al., 2015). To overcome these disadvantages, epNTR was generated to increase the enzymatic activity and sensitivity of MTZ via zebrafish codon optimization and changes in a few amino acids (Tabor et al., 2014). The epNTR has been used to examine neuronal function and regeneration of tendons and muscles in zebrafish (Niu et al., 2020). In the present study, Ins:epNTR-mCherry model that presented highly efficient ablation of pancreatic β-cells following the administration of a low-dose MTZ was developed using epNTR, thereby creating a novel DPN model that facilitates the visualization of the damage to nerve endings in the PLLn, without inducing the off-target effects associated with MTZ.

Hyperglycemia induces excess ROS production by upregulating glycolysis in the mitochondria, wherein the electron transport chain is overloaded. The highly active polyol pathway is involved in aldose reductase-mediated glucose metabolism, which can lead to cellular damage such as glucose toxicity (Lin et al., 2023). In a previous study, epalrestat, an aldose reductase inhibitor, exhibited protective effects against DPN by alleviating ROS production in an STZ-induced mouse model. Combined treatment with epalrestat and alpha-lipoic acid has further demonstrated beneficial effects in patients with DPN (Ramirez and Borja, 2008; Li et al., 2016; Zhao et al., 2018), suggesting that excess ROS production is the main cause of DPN. Although the glucose levels in Ins:epNTR-mCherry zebrafish developed in the present study were 2.5-fold higher compared with those in the normoxic group, no changes were observed in ROS levels in the PLLg. This finding suggested that the observed increase in glucose levels, indicative of hyperglycemia, was insufficient to trigger ROS production in the Ins:epNTR-mCherry model, therefore proposing the use of Ins:epNTR-mCherry as an insulinopenia model. Insulin plays a major role in glucose metabolism by regulating glucose uptake, breakdown, and synthesis via glycolysis and gluconeogenesis in insulin-dependent cell types, such as those in the liver, muscle, and adipose tissue (Pessin and Saltiel, 2000). Furthermore, insulin acts as a neurotrophic factor through its effects on IR signaling in the PNS. Insulin supplementation enhanced the neurite outgrowth of sympathetic and dorsal root ganglion (DRG) sensory neurons from chick embryos in primary cultures and in an adult rat model in a dose-dependent manner (Recio-Pinto et al., 1986). In addition, it promotes neurite regrowth in dissociated DRG neurons (Fernyhough et al., 1993). The stimulation of IR signaling via insulin supplementation further upregulates axonal outgrowth in cultured adult sensory neurons thorough the robust activation of the PI3-K/Akt pathway (Huang et al., 2005). These findings indicate that insulin acts as a neurotrophic factor by acting on IR signaling in peripheral sensory neurons. A reduction in the number of pS6-expressing PLLg cells was observed in the Ins:epNTR-mCherry zebrafish in the present study, owing to the ablation of the pancreatic β-cells without inducing any changes in the ROS levels in PLLg (Figures 4, 6). This finding indicates that insulin ligand depletion reduces mTOR activity, which is a downstream target of IR signaling for synapse formation (Agostinone et al., 2018). Furthermore, treatment with BMS-754807, an inhibitor of IGF-1 and an insulin receptor identical to the insulin ligand, induced nerve defects in the PLL, corresponding to those induced by insulin depletion (Figure 5). This finding indicates that loss of IR signaling causes peripheral neuropathy in the PLL of Ins:epNTR-mCherry zebrafish. Thus, IR signaling plays a key role in inducing DPN in Ins:epNTR-mCherry zebrafish. The findings of this study are supported by those of several previous studies. In one previous study, euglycemic rats with no insulin production due to STZ-induced ablation displayed mechanical hyperalgesia, a representative sign of DPN (Romanovsky et al., 2010). Furthermore, in another study, Goto–Kakizaki rats with impaired glucose tolerance and overt insulinopenia exhibited reduced nerve conduction velocity and loss of unmyelinated axons, consistent with the general features of DPN (Murakawa et al., 2002). This suggests that insulin deficiency, even in the absence of hyperglycemia, causes DPN. As the increase in glucose levels was insufficient to trigger ROS production as the primary cause of DPN in the Ins:epNTR-mCherry model, we suggest that the developed Ins:epNTR-mCherry model serves as a DPN model, wherein DPN was induced by insulin deficiency rather than hyperglycemia.

This study demonstrated that the loss of IR signaling, which affects neuronal synapse formation, is one possible mechanism underlying DPN. IR signaling is involved in the regulation of excitatory and inhibitory receptor trafficking for postsynaptic neurotransmission in the central nervous system (Passafaro et al., 2001; Skeberdis et al., 2001). Chiu et al. further reported that IR signaling plays an essential role in the maintenance of synaptic density and branching involved in the visual circuit from postsynaptic retinotectal neurons in living *Xenopus* (Chiu et al., 2008). We further demonstrate that the depletion of IR signaling results in the synaptic loss and reduction of axon bifurcation in the PLLn, followed by defective rheotaxis behavior (Figure 3); thus, synaptic loss due to impaired IR signaling may lead to DPN development in Ins:epNTR-mCherry zebrafish.

## Data Availability

The raw data supporting the conclusions of this article will be made available by the authors, without undue reservation.

## Supplementary material

Supplementary Figure 1 Ins:epNTR-mCherry expressing *Tg (tubb2b:dsred)* larvae showed discrete expression of fluorescent proteins emitting the same red colors. **(A,B)** Lateral views of the trunk of the *Tg (tubb2b:dsred)* **(A)** and Ins:epNTR-mCherry expressing *Tg (tubb2b:dsred)* larvae **(B)** at 3dpf. The arrow indicates pancreatic β-cells. Scale bar, 200 μm in **(A)**.

Supplementary Movie 1 Depletion of insulin production decreases the performance of rheotaxis behavior in Ins:epNTR-mCherry larvae. Transgenic larvae treated with DMSO (top lane) and siblings treated with MTZ (middle lane) exhibited normal rheotactic behavior, displaying counter-flow swimming against the direction of water flow. Transgenic larvae treated with MTZ (bottom lane) exhibited impaired rheotactic behavior and displayed no bias in the direction of water flow.
