# Supplementary figures and images for: Transgenic zebrafish as a model for investigating diabetic peripheral neuropathy: investigation of the role of insulin signaling
Source: Front Cell Neurosci. 2024 Sep 24;18:1441827. doi: 10.3389/fncel.2024.1441827 (PMC11458509; doi:10.3389/fncel.2024.1441827)

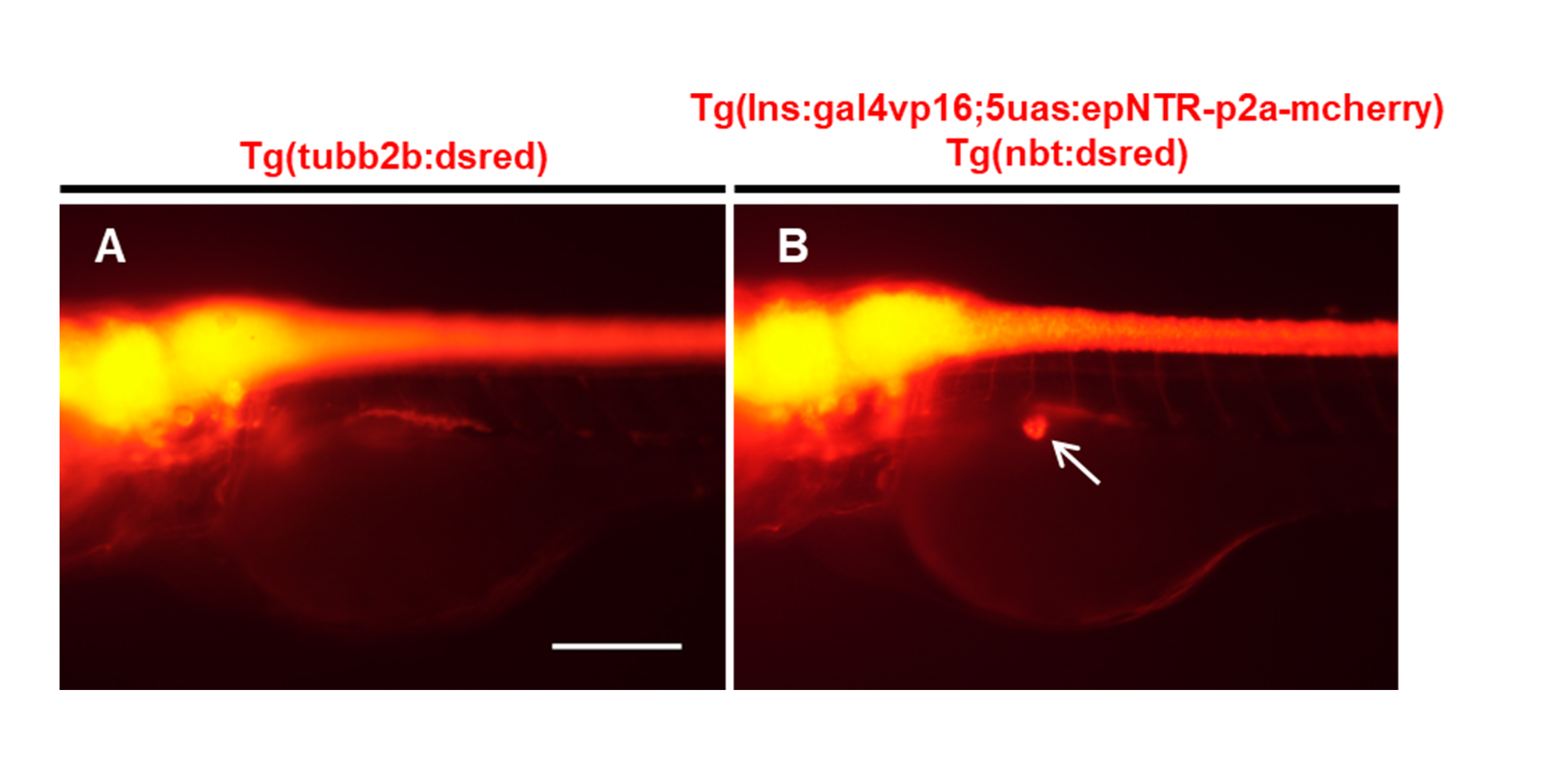

Supplement: Supplementary Figure 1 — Ins:epNTR-mCherry expressing Tg (tubb2b:dsred) larvae showed discrete expression of fluorescent proteins emitting the same red colors. (A,B) Lateral views of the trunk of the Tg (tubb2b:dsred) (A) and Ins:epNTR-mCherry expressing Tg (tubb2b:dsred) larvae (B) at 3dpf. The arrow indicates pancreatic β-cells. Scale bar, 200 μm in (A). [file Image_1.TIF]
